# Profiling the persistent and episodic nature of long COVID symptoms and the impact on quality of life and functional status: a cohort observation study

**DOI:** 10.7189/jogh.15.04006

**Published:** 2025-02-07

**Authors:** Rebecca Owen, Ruth EM Ashton, Tom Bewick, Robert J Copeland, Francesco V Ferraro, Clare Kennerley, Bethan E Phillips, Thomas Maden-Wilkinson, Thomas Parkington, Lindsay Skipper, Callum Thomas, Ross Arena, Federico Formenti, Cemal Ozemek, Sundar Kumar Veluswamy, Rachita Gururaj, Mark A Faghy

**Affiliations:** 1 Biomedical and Clinical Science Research Theme, School of Human Sciences, University of Derby, Derby, UK; 2 Research Centre for Physical Activity, Sport and Exercise Sciences (PASES), Institute of Health and Wellbeing (IHW), Coventry University. Coventry, UK; 3 Department of Respiratory Medicine, University Hospitals of Derby and Burton NHS Foundation Trust, Uttoxeter Road, Derby, UK; 4 Physical Activity, Wellness and Public Health Research Group, School of Sport and Physical Activity, Sheffield Hallam University, Sheffield, UK; 5 School of Medicine, University of Nottingham, Nottingham and Derby, UK; 6 Patient and Public Involvement and Engagement Representative, Derby. UK; 7 Department of Physical Therapy, College of Applied Health Sciences, University of Illinois at Chicago, USA; 8 Centre for Human and Applied Physiology, King’s College London, London, UK; 9 Department of Physiotherapy, Ramaiah Medical College, Bengaluru, India

## Abstract

**Background:**

Post-viral issues following acute infection with coronavirus disease 2019 (COVID-19), referred to widely as long COVID, are associated with episodic, persistent, and disabling symptoms affecting quality of life and functional status. Evidence demonstrates a significant impairment and long disease course, but there remains limited empirical data to profile and determine the fluctuating symptom profile of long COVID.

**Methods:**

We devised a 16-week, multicentre prospective cohort observation study to profile changes in patient-reported outcomes, and biological, physiological, psychological, and cognitive parameters following diagnosis and/or referral to an established long COVID clinic. Following baseline assessments, participants completed four face-to-face visits interspersed with telephone consultations. Face-to-face visits included physiological assessment, patient-reported outcome measures (PROMs), functional status, and respiratory function. Telephone consultations involved PROMs and symptom profiling.

**Results:**

Patient-reported outcomes improved from baseline to week sixteen, but demonstrated between visit fluctuations in frequency and severity. Further findings highlight the severity and frequency of long COVID symptom profiles and the extent of quality of life and functional status impairment.

**Conclusions:**

The data presented here highlight the episodic and relapsing nature and should be used to help characterise long COVID disability. They can inform the development of long COVID-specific guidelines and support services that can adequately respond to the reductions in patient well-being.

Symptoms of acute viral infections that persist in the weeks, months, and years’ post-infection are collectively referred to as post-viral illnesses. The most devastating epidemic in recorded history was the 1918 Spanish Flu epidemic, with an estimated global mortality between 24–50 million people over three distinct waves of infection [1]. Of particular interest was the high prevalence of reported complications and impaired recovery, with physical exertion and fatigue being documented as important limiting factors [2]. More recent epidemics, including those of severe acute respiratory syndrome coronavirus (SARS-CoV) or severe acute respiratory syndrome coronavirus 1 (SARS-CoV-1) (2002–04), have also demonstrated persistent symptoms that impact functional status and quality of life with evidence showing sustained impact at 12 months post-infection [3]. This is also true for the severe acute respiratory syndrome coronavirus 2 (SARS-CoV-2) COVID-19 virus that arose in 2019 and went on to be transmitted globally, leading to >771 million reported cases and >6 million deaths [4]. The actual figures are likely to be much higher due to the time required to develop and provide access to testing, which has subsequently been removed as part of the world's approach to living with COVID-19.

Post-viral complications following acute infection with COVID-19, referred to as post-acute COVID syndrome or more widely as long COVID, are associated with persistent and often disabling symptoms that affect individuals’ quality of life and functional status [5]. A lack of consistency in clinical definitions and implementation of appropriate reporting methods, together with a dearth of pathophysiological and mechanistic understanding, make it difficult to provide accurate estimations of those living with long COVID. It has been suggested that one in ten people experience persistent symptoms that are not resolved at 12 months following a COVID-19 infection, with global trends indicating that it affects 65–150 million people worldwide [6,7]. Accordingly, there are currently no definitive curative treatments for patients with long COVID. Although some clinician-initiated treatments appear promising, they have not undergone rigorous testing in controlled clinical trials.

In response to the emerging narrative of persistent and debilitating symptoms in long COVID, a series of studies were established to quantify patient outcomes and pathophysiologic function over time. Cohort observation study designs are commonplace in clinical research settings to identify and evaluate causes, risks, or changes in diseases or health-related events. In doing so, they can adopt a prospective or retrospective approach. Retrospective cohort designs have been widely implemented and make use of existing data sets that are recorded in clinical settings to determine the long-term outcomes for patients in specific clinical areas. In the context of long COVID, Taquet et al. [8] conducted a retrospective cohort study via electronic health records data from >81 million patients, including 273 618 COVID-19 survivors. They found that 57% had at least one feature of long COVID during the six-month study period, which was not resolved at 12 months in 37% of cases. The most reported symptoms included abnormal breathing (18%), fatigue/post-exertional malaise (13%), chest/throat pain (13%), headache (9%), other pain (12%), abdominal symptoms (16%), myalgia (3%), cognitive symptoms (7%), and anxiety/depression (23%). While it is recognised that this methodology allows a fast analysis of large data sets and for conclusions to be derived quickly, they are limited and cannot be used to establish definitive causality in chronic disease. Additionally, such retrospective approaches are not designed to support closer inspection and determination of regular fluctuations in symptom profiles and the ongoing persistence of clinical features that affect everyday life.

The use of prospective cohort observations has also produced intentionally-designed data that has been used to increase knowledge of risk factors and patient outcomes over a period following infection with COVID-19. The nature and design of prospective studies permit insight over prolonged periods from a clinical perspective, where data can be collected and analysed about important health and well-being outcomes about prognosis and to evaluate the efficacy of interventions. Evidence to date demonstrates significant impairment and a long disease course (>12 months), but there remains little insight into the episodic and debilitating nature of long COVID, which is prone to exacerbation. Accordingly, we designed this study to profile the frequency and variations in the patient-reported outcomes, as well as biological, physiological, psychological, and cognitive parameters in the 16 weeks following a confirmed diagnosis and/or referral to an established long COVID clinic using a mixed-methods approach.

## METHODS

Following institutional (ETH2021-3135) and National Health Service (NHS) ethical approval (IRAS ID: 292920), we conducted a 16-week prospective observation cohort study at the University of Derby and Sheffield Hallam University, UK. Data collection started in June 2020 and finished in May 2023.

### Recruitment, screening, and eligibility

Patients who were hospitalised because of severe COVID-19 during the acute phase and then developed long COVID were assessed according to eligibility criteria and recruited directly from Derbyshire Community Health Services and Sheffield Teaching Hospitals NHS Foundation Trust. Long COVID patients were also assessed according to the eligibility criteria and were recruited following referral/contact with a long COVID clinic or as having suspected or confirmed long COVID. Social media and targeted recruitment from established pages were used to advertise the opportunity to engage with the trial.

We included participants scoring two or more on the post-COVID-19 Functional Status Scale (PCFS) [9] and having persistent symptoms consistent with long COVID according to the World Health Organization (WHO) definition [10] who were over 18 years of age and were able to understand verbal or written information in English. We excluded those who did not meet this inclusion criteria and/or had reduced or lack of mental capacity.

### Experimental protocol

We profiled the determinants of recovery using a mixed-method approach. Participants attended five face-to-face visits each occurring approximately four weeks apart, interspersed by biweekly telephone calls (**Table 1**). On each face-to-face visit, we collected their physiological variables and patient-reported outcome measures (PROMs), and we conducted functional status tests (6-Minute Walk Test (6MWT) and Timed Up and Go (TUG)) and respiratory function tests. During telephone consultations, PROMs and symptom profiling were completed and details of contact with healthcare services were taken.

**Table 1:** Experimental protocol

**Week 0**	Study enrolment. Visit 1 (baseline): background and medical history (occupation, prior COVID-19 health, route into study, smoking history), blood sampling, anthropometry, symptom reporting, physiological measures (respiratory and cardiovascular), functional status, and PROMs. Approximately 120 min.
**Week 2**	Telephone consultation 1: healthcare contact, symptom reporting, and PROMs (exc. MOCA). Approximately 20–30 min.
**Week 4**	Visit 2: Symptom reporting, physiological measures (respiratory and cardiovascular), functional status, and PROMs. Approximately 90 min.
**Week 6**	Telephone consultation 2: healthcare contact information, symptom reporting, and PROMs (exc. MOCA). Approximately 20–30 min.
**Week 8**	Visit 3: symptom reporting, physiological measures (respiratory and cardiovascular), functional status, and PROMs. Approximately 90 min.
**Week 10**	Telephone consultation 3: healthcare contact information, symptom reporting, and PROMs (exc. MOCA). Approximately 20–30 min.
**Week 12**	Visit 4: symptom reporting, physiological measures (respiratory and cardiovascular), functional status, and PROMs. Approximately 90 min.
**Week 14**	Telephone consultation 4: healthcare contact information, symptom reporting, and PROMs (exc. MOCA). Approximately 20–30 min.
**Week 16**	Study completion. Visit 5: symptom reporting, physiological measures (respiratory & cardiovascular), functional status, and PROMs. Approximately 90 min.

MOCA – Montreal Cognitive Assessment, PROMS – patient-reported outcome measures, exc. - except

### Baseline visit

Following screening, we collected anthropometric data on height and weight, date of birth, sex, past medical history, smoking history, and occupational status, as well as details regarding admission and contact with primary and secondary care for those patients who had been hospitalised due to either acute or long COVID-related symptoms. A venous blood sample was taken from the antecubital fossa region of the arm, allowing for the measurement of inflammatory and metabolic markers (full blood count, red blood cells, white blood cells, haemoglobin, haematocrit, mean corpuscular volume, mean corpuscular haemoglobin, mean corpuscular haemoglobin concentration, red cell distribution width, platelets, neutrophils, lymphocytes, eosinophils, monocytes, basophils, ferritin, D-dimer, C-reactive protein, lactate dehydrogenase) [11–17].

### Scales

We applied the PCFS, the EuroQol-5 Dimension-5 Level (EQ-5D-5L), the Medical Research Council (MRC) Dyspnoea Scale, the Fatigue Assessment Scale (FAS), the Modified Fatigue Impact Scale (MFIS), and the Montreal Cognitive Assessment (MoCA) scales to the patients and assessed their symptoms profile.

The PCFS was developed to assess recovery following COVID-19 infection, covering the entire range of functional limitations, such as changes in lifestyle and social activities [9]. The scale determines how much an individual is affected in their everyday life by COVID-19, from having no limitations (0) to suffering from severe limitations in everyday life, without being able to care for themselves and being dependent on nursing care and/or assistance from another person due to symptoms, pain, depression and anxiety (4).

The EQ-5D-5L is a commonly used assessment for quality of life, comprising five dimensions: mobility, self-care, usual activities, pain/discomfort, and anxiety/depression [18]. Each dimension has five levels: no problems, slight problems, moderate problems, severe problems, and extreme problems. A visual analogue scale is used to record the patient’s self-rated health, with endpoints being ‘the best health you can imagine’ and ‘the worst health you can imagine’.

The MRC Dyspnoea Scale is a valid method used to assess the degree to which dyspnoea affects functional ability on a scale of 0–4 [19]. The scale measures perceived respiratory disability, allowing patients to indicate the extent of breathlessness on their mobility.

The FAS is a 10-item self-report scale evaluating symptoms of fatigue. The FAS treats fatigue as a unidimensional construct, measuring both physical and mental symptoms [20]. The total score ranges from 10–50, with a higher score accounting for more severe fatigue. A total score of <22 indicates a healthy level of fatigue, 22–34 mild-to-moderate fatigue, and ≥35 severe fatigue.

The MFIS is a 20-item self-reported questionnaire assessing fatigue, consisting of nine ‘physical’, 10 ‘cognitive’, and two ‘psychosocial’ items [21]. Higher scores indicate a greater impact of fatigue on quality of life and are calculated for each subscale (physical: 0–36; cognitive: 0–40; psychosocial: 0–8) with a maximum total score of 84 [21].

The MoCA is a widely used assessment in clinical settings and research. It is a validated, highly sensitive measure used for the early detection of mild cognitive impairment, assessing short-term memory, visuospatial abilities, executive functions, attention concentration and working memory, language, and temporal and spatial orientation [22]. Two distinct versions of the MoCA were used as recommended to reduce the impact of the learning effect.

Patients reported and described symptoms and the impact these have on daily life on a scale of 0–10. The symptom score measure was also completed, detailing the severity of symptoms for the previous 24 hours.

### Functional status

#### 6MWT and TUG

The 6MWT is a standardised and widely-used measure of functional status which also allows for the assessment of responses to interventions and the prediction of morbidity and mortality [23–25]. Here we conducted it to 2002 American Thoracic Society guidelines [26], whereby we instructed the participant to walk up and down the corridor, covering the greatest distance possible over six minutes.

The TUG is a reliable measure accepted for use across multiple clinical populations and is validated as a predictor of frailty and risk of falls in elderly adults [27]. We instructed participants to stand from a seated chair with armrests and walk to and from a three-meter marker, where they were required to tap the practitioner’s hand and sit back down [28]. A total of three attempts were timed, with the quickest recorded as the best effort.

### Physiological measures

We measured blood oxygen saturation using a Nonin Medical Pulse Oximeter (Model 2500, Nonim Medical, INC., Plymouth, MN, USA), resting heart rate and blood pressure using an automatic blood pressure monitor (Omron M2, Omron Healthcare Co Ltd, Kyoto, Japan) and core body temperature via a tympanic reading using a Braun thermometer (Braun Thermoscan model 6022, Germany).

#### Lung and respiratory muscle function

We recorded the patients’ maximum inspiratory pressure (MIP) and maximum expiratory pressure (MEP) measurements on face-to-face visits according to published guidelines [29]. Specifically, we assessed MIP using a handheld respiratory pressure meter (RP Check, MD Diagnostics Ltd, Maidstone, UK) with an occluded nasal pathway. Manoeuvres were initiated from residual volume and a maximal inspiratory effort was maintained for three seconds. We similarly assessed MEP using the same handheld device; however, participants initiated the manoeuvre from total lung capacity followed by a maximal expiration maintained for three seconds. The best of three consecutive values within either 10% or, if lower, 10 cm H_2_0 was taken as the values for MIP and MEP. However, if this condition was not met, we took the average of the three highest values from ten efforts as the values [29].

We used a handheld, electronic spirometer (SpiroConnect, MedChip Solutions Ltd, Kent, UK) to measure forced vital capacity (FVC), forced expiratory volume in one second (FEV1), the FEV1:FVC ratio, and peak expiratory flow (PEF) with an occluded nasal pathway while seated. We took manoeuvres according to appropriate guidelines [30] and initiated them from total lung capacity. A maximal expiratory effort was maintained for five seconds; a minimum of three attempts were performed with an acceptability criterion being when there was a ≤0.150 L differences between the largest and next largest FVC and FEV1 measurements [31]. Breathing rate was assessed while seated at rest by observing participants' chest rise and fall over a 10-second period, which was then extrapolated to provide a one-minute breathing rate.

### Data analysis

We transferred the raw data from the case report form (CRF) into Microsoft Excel, version 16.92 (Microsoft Corporation, Redmond, Washington, USA), after which we imported them into Python, version 3.11.5, through the ‘pandas’ package, version 2.0.3. We then generated time-plot and heat map figures using ‘seaborn’, version 0.12.2 and ‘matplotlib’, version 3.7.2. We also imported the data set into SPSS, version 29.0.1.1 (IBM, Armonk, New York, USA) to determine descriptive statistics (*i.e.* means (x̄) and standard deviations (SDs) or medians (MDs) and interquartile ranges (IQRs)) and the distribution of the data, draw box plots, and conduct Mauchly’s sphericity test, one-way repeated measures analysis of variance (RMANOVA), and *post hoc* analyses. Due to its robustness, we used RMANOVA where the assumption of normality was not met, but sphericity was assumed [32]. In line with the literature [33], we used multiple imputation (MI) in cases where 5–10% of data were missing, whereby we used the MI model in SPSS to replicate the incomplete data set five times and replace the missing data in each replicate with plausible values. We calculated a single MI by combining the estimates obtained from each completed data set and pooling the data according to Rubin’s rules [34,35]. We also used MIfor missing data for those who did not reach the end of the study, but had completed >2 face-to-face visits (n = 8) [33,36]. However, we did not use it in cases where worsening symptomology resulted in a participant being unable to perform a measure. Lastly, we used normative data and expected values for comparison to this cohort.

## RESULTS

A total of 75 participants met the WHO clinical definition of long COVID [10] (**Table 2**). The mean time from initial infection to the date of participation was one year and two months. Seven participants were hospitalised during acute COVID-19 infection, with length of stay ranging from 1–32 days. These patients then went on to experience long COVID, as diagnosed according to the WHO definition [10]. At the time of the baseline visit, 99% were vaccinated with 54.7% receiving three doses. One or more comorbidities were experienced by 89% of participants, and 73.3% of participants were non-smokers.

**Table 2:** Participant characteristics (n = 75)*

Demographics	
Females	49 (65)
Age in years, mean (SD)	48 (12)
Smoking	
*Non-smoker*	54 (73.3)
*Previous smoker*	20 (28.8)
*Smoker*	1 (1.3)
**Vaccination status**	
Vaccinated	74 (99)
*One dose*	5 (7)
*Two doses*	23 (30)
*Three doses*	41 (55)
*Four doses*	6 (8)
Pre-LC	40 (53)
Post-LC	26 (35)
**Comorbidities**	
Endocrine	11 (15)
Renal	6 (8)
Cardiovascular	21 (28)
Gastrointestinal	34 (45)
Neurological/cerebrovascular	21 (28)
Malignancy (including haematological)	8 (11)
Other	8 (11)
One comorbidity	14 (19)
Two comorbidities	22 (29)
Three or more comorbidities	27 (36)

*Values presented as n (%) unless specified otherwise.

### Long COVID symptoms

**Figure 1:**
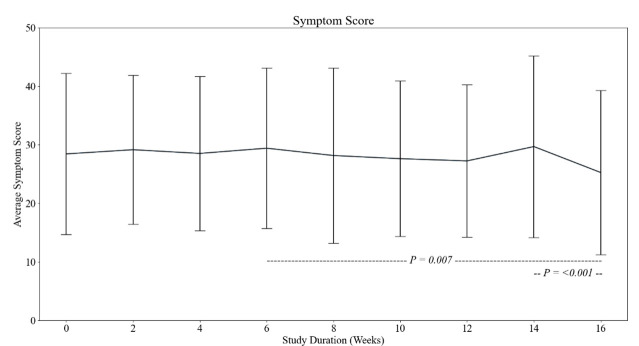
Change in symptom scores across each time point Hashed lined and *P*-values represent significant changes between highlighted time points.

**Figure 2:**
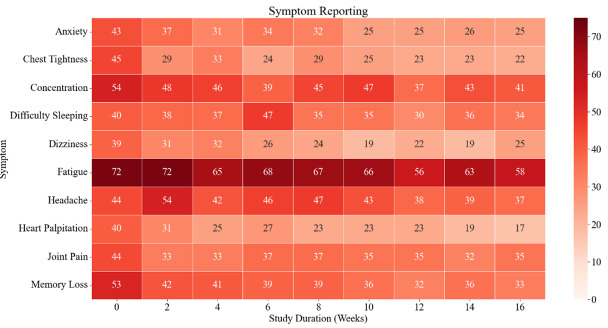
Symptom profiling across the duration of the study, colour-coded to indicate severity and derived from the symptom burden questionnaire.

The cumulative symptom score relative to severity was an arbitrary unit (AU)  of 28 (SD = 14) at baseline. The *post hoc* analysis showed statistically significant differences between week six (AU = 29 (SD = 14)) and week 16 (AU = 25 (SD = 14); *P* = 0.007), as well as week 14 (AU = 30 (SD = 16)) and week 16 (*P* < 0.001) (**Figure 1**). Fatigue was the most reported symptom across the 16 weeks, followed by difficulty concentrating. The prevalence of other symptoms varied over the 16 weeks, but consisted of headaches, difficulty sleeping, and cognitive disturbance (**Figure 2**).

#### PROMs

**Figure 3:**
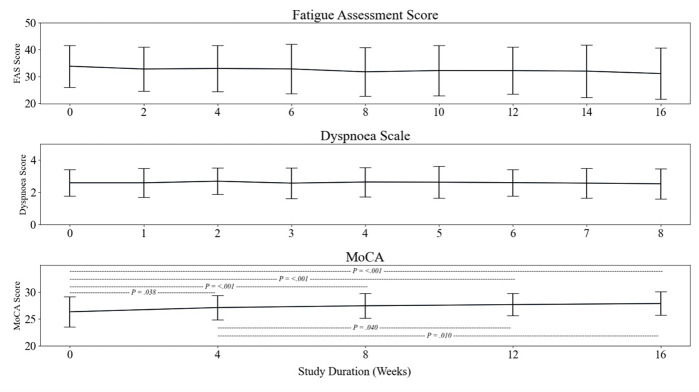
Panel plot demonstrating persistence of fatigue, breathlessness, and cognitive function throughout the study. Hashed lines and *P*-values represent significant changes between highlighted time points.

**Figure 4:**
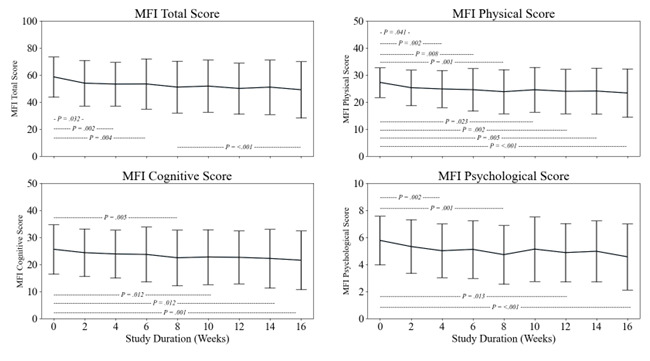
Change in each domain of the MFIS, across the study. Hashed lines and *P*-values represent significant changes between highlighted time points.

Cognitive function improved from baseline (AU = 23 (SD = 9)) to week 4 (AU = 27 (SD = 2); *P* = 0.038), week 8 (AU = 27 (SD = 2); *P* < 0.001), week 12 (AU = 28 (SD = 2); *P* < 0.001), and week 16 (AU = 28 (SD = 2); *P* < 0.001). There were further improvements between week 4 and week 12 (*P* = 0.040) and week 16 (*P* = 0.010) (**Figure 3**). Dyspnoea was at an AU of 3 (SD = 1) at baseline and was unchanged at any time point. FAS indicates severe fatigue at baseline (AU = 34 (SD = 9)) with an improvement, whereby each week the FAS score was reduced to mild-moderate fatigue with a global significance of *P* = 0.034. This trend fluctuated between time points, with a final AU score of 31 (SD = 10). Fatigue was further assessed with the MFIS, and the cumulative score at baseline (AU = 59 (SD = 15)) followed a similar trend to FAS, with levels of fatigue fluctuating across the 16-weeks. There was a significant change from baseline to week 2 (AU = 54 (SD = 17); *P* = 0.032), week 4 (AU = 53 (SD = 16), *P* = 0.002), week 6 (AU = 53 (SD = 19); *P* = 0.004), and week 8 (AU = 51 (SD = 18)) to week 10 (AU = 52 (SD = 19); *P* < 0.001). When analysed for each subsection of the MFIS. When analysed for each subsection of the MFIS (**Figure 4**), physical fatigue had a significant improvement from baseline (AU = 28 (SD = 5)) to week 2 (AU = 25 (SD = 7); *P* = 0.041); week 4 (AU = 25 (SD = 7); *P* = 0.002) week 6 (AU = 25 (SD = 8); *P* = 0.008), week 8 (AU = 24 (SD = 8); *P* < 0.001), week 10 (AU = 25 (SD = 8); *P* = 0.023), week 12 (AU = 24 (SD = 8); *P* = 0.002) week 14 (AU = 24 (SD = 8); *P* = 0.005), and week 16 (AU = 23 (SD = 9); *P* < 0.001). Cognitive fatigue was assessed at an AU of 26 (SD = 9) at baseline and followed a similar trend of improvement from baseline to week 8 (AU = 22 (SD = 0); *P* = 0.005), week 10 (AU = 22 (SD = 10); *P* = 0.012), week 14 (AU = 22 (SD = 11); *P* = 0.012), and week 16 (AU = 22 (SD = 11); *P* < 0.001). Psychosocial fatigue followed a similar trend, improving from baseline to week 4 (AU = 5 (SD = 2); *P* = 0.002), week 8 (AU = 5 (SD = 1); *P* < 0.001), week 12 (AU = 5 (SD = 2); *P* = 0.013) and week 16 (AU = 5 (SD = 2); *P* < 0.001).

#### Quality of life

**Figure 5:**
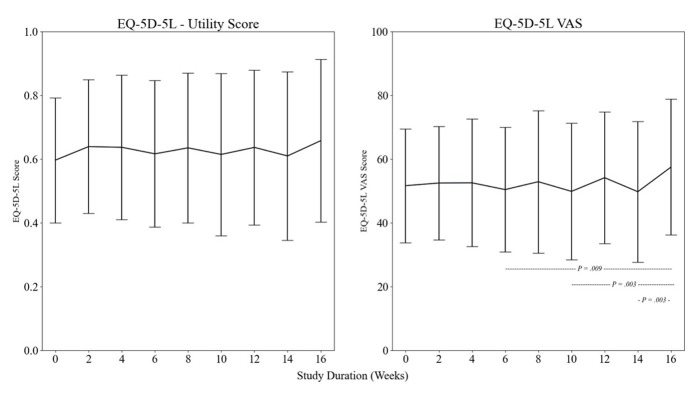
Reported impact upon quality-of-life using the EQ-5D-5L and the EQ-5D-5L VAS score, hashed lined and *P*-values represent significant changes between highlighted time points.

Across the 16 weeks, the mean utility index score for the EQ-5D-5L ranged from 0.002–1 but did not significantly change between time points (**Figure 5**). The mean EQ visual analogue scale improved between week 6 (AU = 50 (SD = 20)) and week 16 (AU = 57 (SD = 20); *P* = 0.009), week 10 (AU = 50 (SD = 21)) and week 16 (*P* = 0.003), and week 14 (AU = 50 (SD = 21)) to week 16 (*P* = 0.003).

#### Functional status

**Figure 6:**
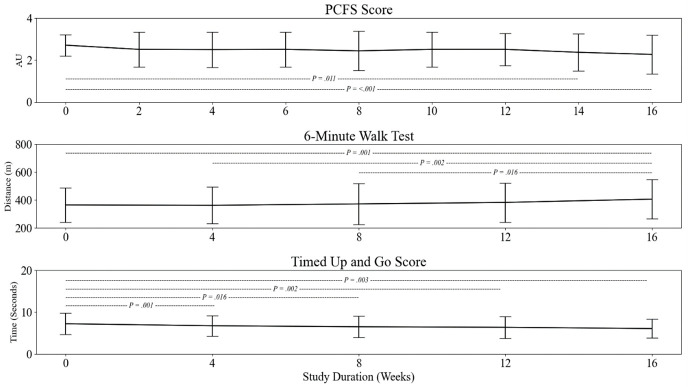
Panel plot demonstrating impaired functional status assessed by the PCFS, 6MWT, and the TUG. Hashed lines and *P*-values represent significant changes between highlighted time points.

PCFS at baseline was at an AU of 2.7 (SD = 0.5) and improved relative to week 16 (AU = 2.3 (SD = 0.9); *P* = <0.001) and week 14 (AU = 2.4 (SD = 0.9); *P* = 0.011). The 6MWT score at baseline was 365 m (SD = 123) and was subsequently improved between baseline and week 16 (406 m (SD = 141); *P* = <0.001) (**Figure 6**). *Post hoc* analysis also showed fright further improvements between week 4 and week 16 (*P* = 0.002); and finally, week 8 and week 16 (*P* = 0.018). TUG was improved between baseline (AU = 7.2 (SD = 2.5 seconds)) and week 4 (6.7 seconds (SD = 2.4); *P* < 0.001) and baseline to week 8 (AU = 6.5 (SD = 2.6 seconds); *P* = 0.016) between baseline and week 12 (6.3 seconds (SD = 2.6); *P =* 0.002) and between baseline and week 16 (6 seconds (SD = 2.2); *P* = 0.003). There were no other between-time point changes.

#### Physiological measures

**Figure 7:**
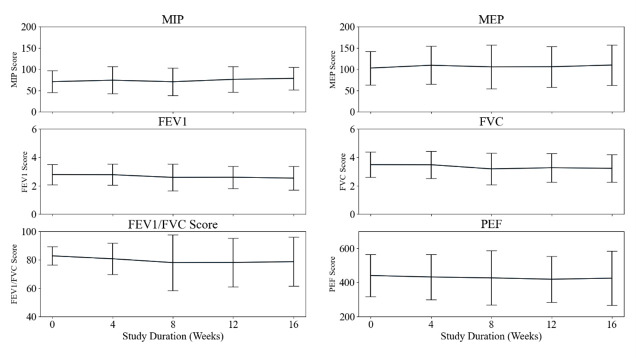
Panel plot profiling inspiratory and expiratory muscle strength and lung function data throughout each face-to-face visit. Hashed lines and *P*-values represent significant changes between highlighted time points.

MIP at baseline was at an AU of 71 cm H2O (SD = 26) and was improved between baseline and week 16 (AU = 79 cm H2O (SD = 28); *P* = 0.015). There was no significant change between any other time points for MIP or MEP. The global effect was significant for FEV1 (*P* = 0.003), FEV1/FVC (*P* = 0.045), and FVC (*P* < 0.001) (**Figure 7**). However, *post hoc* analysis showed no significance within pairwise comparisons. There was no significant difference in PEF across the 16 weeks. Blood panel results for a subset of 44 participants (**Table 3**) were derived from routine clinical investigations with means and standard deviations from the cohort analysis being largely considered as within normal ranges. However, individual reporting of minimum and maximum values showed consistent upregulation of some markers (WBC, MCV, MCH, RDW, platelets, neutrophils, eosinophils. Monocytes, basophils, ferritin, D-dimers, CRP), indicating some biochemical disturbance/irregularities.

**Table 3:** Blood panel results (n = 44)*

	Expected values	x̄ (SD)	Minimum	Maximum
WBC × 10^9^/L	4.3–11	7.29 (1.99)	4.10†	13.29†
RBC × 10^12^/L	4.2–6.9	4.68 (0.54)	2.83	5.77
Haemoglobin in g-L	Males: 130–180; females: 120–160	134.52 (16.67)	67.00	169.00
Haematocrit in %	Males: 40–50; females: 36–48	41 (4)	22	50
MCV (fL)	80–100	86.99 (7.30)	65.10†	102.40†
MCH (pG)	27–32	28.84 (2.93)	19.80†	33.70†
MCHC (g-L)	320–360	331.05 (11.97)	300.00†	352.00
RDW (%)	11.5–14.5	13.01 (1.30)	11.70	17.80†
Platelets ×10^9^/L	150–400	298.36 (57.52)	200.00	439.00
Neutrophils (%)	1.8–7.8	4.48 (1.67); 60.55 (8.22)	2.35; 40.4	10.58; 79.6†
Neutrophils ×10^9^/L				
Lymphocytes (%)	0.7–4.5	2.09 (0.67)	1.15	4.42
Lymphocytes × 10^9^/L		29.27 (7.32)	13.50	45.30
Eosinophils (%), × 10^9^/L	0.0–0.4	0.13 (0.08)	0.01; 0.1	0.49†
Eosinophils × 10^9^/L		1.67 (0.87)		4.2†
Monocytes (%)	0.1–1.0	0.57 (0.15)	0.33; 4.3	1.09†
Monocytes × 10^9^/L		8.00 (1.87)		13.3†
Basophils (%)	0.0–0.2	0.03 (0.01)	0.01	0.07
Basophils × 10^9^/L		0.41 (0.22)	0.10	1.30
Ferritin (ug-L)	Males: 30–300; females: 10–200	92.01 (100.15)	0.98†	430.00†
D-dimers (ug-mL)	0.0–0.5	0.38 (0.29)	0.00	1.85†
CRP (mg L)	<0.3	2.21 (3.63)	<0.01	19.0
LDH (IU-L)	140–280	177.95 (20.90)	121.00†	207.00

CRP – C-reactive protein, LDH – lactate dehydrogenase, MCH – mean corpuscular haemoglobin, MCHC – mean corpuscular haemoglobin concentration, MCV – mean corpuscular volume, RBC – red blood cells, RDW – red cell distribution width, WBC – white blood cells

*Means and minimum and maximum values are presented with expected/standardised values.

†Data with a maximum or minimum value outside of expected values.

## DISCUSSION

The key findings of this prospective cohort observation highlight the severity and frequency of long COVID symptom profiles and how they impair quality of life and functional status via clinically relevant PROMs. The data demonstrates little or no improvement over 16 weeks, while the frequency of contact throughout the study demonstrates the episodic and relapsing nature of long COVID. This finding should be used to help characterise long COVID disability and to inform the development of related guidelines and support services that can adequately respond to the observed reductions in all areas of patient well-being.

To our knowledge, this is the first study to objectively collect biological, physiological, psychological, and cognitive parameters with regular frequency and intensity. It is evident from the data across the patient profiles that performance in all areas of the study was well below expected clinically relevant ranges when compared to existing clinical and normative data sets. Here we provide a multi-dimensional insight into the characteristics/presentation of long COVID, as previous data has been separated by prolonged periods where multiple remissions and changes in patient presentation are reported by patients but not captured. There is evidence of the episodic nature of long COVID, which has been hypothesised in numerous patients' testimonies and accounts [37], but until now has not been demonstrated empirically via cross-sectional methodologies. The undulating/relapsing nature of fatigue, dyspnoea, and symptom profiles includes frequent and intense changes in symptom profiles. Thus, we provide evidence and a need for a distinct characterisation of long COVID patients and their symptoms, but also for personalised intervention approaches.

The burden of symptoms for patients demonstrates little to no progress towards pre-COVID-19 levels, although it is important again to highlight within-sample differences and heterogeneity across the measures and data. Research on long COVID has demonstrated that some, but not all patients improve over time [60]. Still, there remains a level of uncertainty about whether those who are adversely affected by long COVID expect a full recovery and return to pre-long COVID status. This is important when considering the severity of reported disability and organ damage/insults that occur following infection with previous infections with SARs-COV [38] and SARs-COV-2 [39]. In the context of long COVID, a longitudinal cohort study conducted over two years found that only 7.6% (n = 26) of participants fully recovered [40]. Additionally, a multicentre, prospective cohort approach found that of 1170 patients hospitalised with COVID-19, only 29% (n = 239) felt fully recovered and 20% (n = 158) had a new disability six months later [41]. Furthermore, it has been reported that 59.8% of respondents (n = 79) experienced one or more long COVID symptoms in six months following the onset of acute COVID-19, decreasing to 53% at 12 months and increasing to 71.2% at 24 months [42]. In the aforementioned study, the most frequent symptoms at 24 months were fatigue (34.8%), amnesia (30.3%), and concentration difficulties (24.2%), which follows our findings where fatigue, concentration problems, and memory loss were most prevalent across the 16 weeks. These studies highlight the importance of recognising the long-term nature of long COVID, as the knowledge gap of how patients present with high levels of variation demonstrates the need to understand various time points. One study concluded that mild COVID-19 cases lead to a small number of health issues that are resolved within a year of diagnosis and suggests that ‘mild’ cases do not lead to serious or chronic illness for most patients and therefore add only a minor continuous burden to the healthcare system [43]. However, this study did not utilise a long COVID cohort, so the suggestion that individuals will not still be suffering at 12 months is not generalisable to long COVID patients. Long COVID has been labelled the biggest mass-disabling event in history [44], and the aforementioned study fails to acknowledge the struggles of those disabled by their long COVID symptoms. The authors also discussed the frequently reported symptoms associated with long COVID, but also used ‘seriousness’ to quantify risk and did not consider the impact of moderate-severe symptoms on an individual's quality of life.

In line with our findings, previous research has conceptualised long COVID as an episodic illness, which is both multidimensional and unpredictable [37]. Several longitudinal studies adopted methodologies to demonstrate the changes in symptom profiles and functional status from baseline to an end time point (3, 6, 12, 24 months) [8,41–43,45–49]. However, there have been few methodologies that specifically observe and detail what happens between these time points to date; therefore, research regarding the high variation of symptoms beyond one point in time to better understand the episodic nature of long COVID is vital to shaping support services that address the day-to-day challenges that patients experience. The fluctuating symptoms, relapse-remission cycles, and reporting bias may overestimate recovery from long COVID, particularly in studies with shorter follow-up periods or increased time lapses between assessments. The data here supports existing literature that highlights the severity, magnitude, and undulating nature, of symptoms that can reduce the quality of life [50–54]. Findings of health-related quality of life in patients two years post severe COVID-19 infection demonstrate a persistent worsened health status measured by the EQ-5D-5L [55]. In agreement with existing literature [55], the mean utility index score for the EQ-5D-5L for our study was lower compared to population norms at baseline, showing a reduced quality of life [56]. Despite this and other variables significantly improving by week sixteen, we cannot conclude that this signifies recovery due to the nonlinear trajectory and relapsing and remitting nature of long COVID.

The highly cyclical symptom profiles and functional status of long COVID further burden individuals and complicate their ability to plan and engage with typical life, such as reducing individuals' work participation and social activities [57]. Furthermore, the lingering and unpredictable nature of symptoms heavily impacts emotional state and challenges with emotional regulation, increases anxiety, hopelessness, and depression, as well as limits daily functioning [58]. The multidimensional nature of disability and fluctuations of episodic symptoms may vary over a day, and this unpredictability results in participants living and planning for one hour to the next [5].

What is clear is that there remains a significant challenge to address the broad and debilitating symptom profile. The research and findings presented here align with previous research that has identified the most prevalent symptom profiles associated with long COVID and adds greater insight and evidence for characterising long COVID as an episodic and disabling condition by demonstrating the frequent and intense changes that occur in the symptom profile and performance of patients. It has been suggested that patients with chronic diseases will increase their activities when they feel able but with little consideration of the consequences [6]. However, this does not align with our data, which is better associated with the findings of Humphreys et al. [59] who report that long COVID patients prioritise a sense of normality and control over relapse. Our findings indicate that pacing advice of activities seems to have become more widespread and useful through long COVID clinics and television programmes since this work, yet specific guidelines are still scarce. As such, further research is required to document changes in symptom profiles relative to increased volume and intensity of activity.

There remains a dearth of literature that demonstrates efficacy in the form of pharmacological treatments that can be used to treat and address the complex and debilitating long-term outcomes that broadly impact people's lives [60]. Cross-disciplinary discussions among relevant specialists commonly cover complex long COVID cases, yet despite this well-recognised approach, research suggests that its practicality in terms of service utilisation, patient outcomes [61], and patient experience [62] remains equivocal. Furthermore, there are currently no unified strategies in place to support patients with their uncertainties or their daily struggles and reduced quality of life from undulating symptoms. Many patients will benefit from a complex tailored treatment approach, however, identifying patient profiles or phenotyping patients according to their symptom clusters may also present an additional challenge. Symptom clusters have been well-researched and accepted, however, there is limited research regarding the underlying mechanisms behind manifestations [63–67]. Instead of varying pathogenically independent sub-syndromes, research observing sub-phenotypes suggests additive severity of a single, multisystemic, multifaceted post-viral illness [40]. Subsequently, there is a demand to develop approaches to phenotype relative to the underlying pathology and pathophysiology and clustering of symptoms rather than by the symptom presentation. Due to the broad, multi-system, and complex profile of long COVID, assessment and support services have been established that are underpinned by multidisciplinary and integrated care approaches. Considering the evidence for adopting such approaches, there is a need to devise substantive pathways that use coordinated, integrated whole-system thinking approaches [68]. Further assessment tools and protocols are required urgently to inform the development of targeted, patient-centred, interdisciplinary support pathways, to restore functional capacity and quality of life.

A limitation of this research is the limited heterogeneity in the sample, with most participants being Caucasian females. Although the prevalence of self-reported long COVID is the greatest among Caucasian females aged 35–69 years, ethnic minorities have also been adversely affected by the COVID-19 pandemic [69–72]. Further research must also encompass males and young people, including children and young adults [51]. The heterogeneous time points at which patients were included in the study relative to their initial COVID-19 infection may hinder direct comparisons of symptoms and parameters across the study period. However, all participants were recruited in line with the WHO definition of long COVID, and existing research shows that symptoms persist for months and years post-infection but neglects the episodic and undulating nature of symptoms between time points highlighted in this study. Further research including subgroup analyses for comorbidities, vaccination status, and smoking status may be beneficial to understanding symptom trajectory. However, this was beyond the scope of our study, and the sample size and statistical power may have increased the risk of positive and negative findings. Additionally, the sample consists of individuals from a range of functional statuses identified using the PCFS tool. Whilst some participants corresponded to four on the PCFS, those with the most severe symptoms, such as being house/bed bound, would have been unable to complete the study, therefore limiting the generalisability of our results.

## CONCLUSIONS

Here we demonstrate the long-term and broad range of issues affecting people living with long COVID. Due to the increased frequency and intensity of patient contact throughout this study, we highlight the variable and episodic nature of long COVID and the impact that this has on quality of life and functional status. Further research and sustained investment are needed to develop detailed long COVID assessments that can inform targeted, patient-centred, interdisciplinary support pathways which can be used alongside medicinal interventions to restore functional capacity and quality of life.
